# Hepatic Encephalopathy among Patients with Chronic Liver Disease Admitted to the Department of Internal Medicine in a Tertiary Care Centre: A Descriptive Cross-sectional Study

**DOI:** 10.31729/jnma.8206

**Published:** 2023-07-30

**Authors:** Ujjwal Karki, Nischal Upreti, Bindu Gyawali, Sandesh Kumar Shrestha, Chandra Kiran Basnet, Devashish Sharma, Neha Dangol

**Affiliations:** 1 Nobel Medical College Teaching Hospital (P) Ltd., Biratnagar, Morang, Nepal; 2 Curative Service Division, Department of Health Services, Ministry of Health and Population, Teku, Kathmandu, Nepal; 3 Chitwan Medical College and Teaching Hospital, Bharatpur, Chitwan, Nepal; 4 B.P. Koirala Institute of Health Sciences, Dharan, Sunsari, Nepal; 5 Rostov State Medical University, Rostov On Don, Russia; 6 National Hospital and Cancer Research Center, Jawalakhel, Lalitpur, Nepal

**Keywords:** *hepatic encephalopathy*, *liver cirrhosis*, *liver failure*

## Abstract

**Introduction:**

Hepatic encephalopathy is a condition that impairs the neurological and psychiatric function of a patient as a result of advanced liver disease or portosystemic shunt. Early detection and treatment of hepatic encephalopathy can lessen its severity, length of stay in the hospital and potential fatality. The aim of this study was to find out the prevalence of hepatic encephalopathy among patients with chronic liver disease admitted to the Department of Internal Medicine in a tertiary care centre.

**Method:**

A descriptive cross-sectional study was done among patients with chronic liver disease admitted to the Department of Internal Medicine in a tertiary care centre. Data from 1 October 2021 to 15 May 2023 were collected between 20 May 2023 and 30 May 2023 from the hospital records. Ethical approval was obtained from the Institutional Review Committee (Reference number: 808/2023). The diagnosis was made based on the history, clinical examinations, and gradings were done as per West Haven criteria. Convenience sampling was done. Point estimate and 95% Confidence Interval were calculated.

**Results:**

Among 389 patients with chronic liver disease, hepatic encephalopathy was seen in 40 (10.28%) (7.26-13.30, 95% Confidence Interval). The mean age of patients was 55.27±13.52 years.

**Conclusions:**

The prevalence of hepatic encephalopathy among patients with chronic liver disease was lower than the studies conducted in similar settings.

## INTRODUCTION

Hepatic encephalopathy (HE) is a reversible impairment of neurological and psychiatric function found in patients with advanced liver disease or portosystemic shunt.^1^ The prevalence of HE in patients with chronic liver disease was 14.9% among the Nepalese population.^2^

Hepatic encephalopathy accounts for a large number of hospitalizations and readmissions. It has an enormous impact on healthcare and cost.^3,4^ Therefore, it is important to diagnose this condition as the earliest. Minimal HE and Grade I HE are known as covert Hepatic encephalopathy while Grade II, III, and IV are known as overt HE.^5^ Meticulous evaluation of HE in chronic liver disease (CLD) patients is needed to exclude other neurological and psychiatric conditions with similar clinical features.^6^

This study aimed to find out the prevalence of hepatic encephalopathy among patients with chronic liver disease admitted to the Department of Internal Medicine in a tertiary care centre.

## METHODS

A descriptive cross-sectional study was done among patients with chronic liver disease admitted to the Department of Internal Medicine at Nobel Medical College Teaching Hospital, Biratnagar, Morang, Nepal. Data from 1 October 2021 to 15 May 2023 were collected between 20 May 2023 and 30 May 2023 from the hospital records. Ethical approval was obtained from the Institutional Review Committee (Reference number: 808/2023). All chronic liver disease patients admitted to the Department of Medicine for any indication with age >18 years were included in the study. Patients with prior neurological deficits, prior psychiatric conditions interfering with the assessment, and incomplete hospital records were excluded from the study. Convenience sampling was done. The sample size was calculated by using the following formula:


$$\begin{array}{ll} \text{n} & ={\text{Z}}^{2}\times \frac{\text{p}\times \text{q}}{{\text{e}}^{\text{2}}}\\ & =1.96^{2}\times \frac{0.50\times 0.50}{0.05^{2}}\\ & =385 \end{array}$$

Where,

n = minimum required sample size
Z = 1.96 at 95% Confidence Interval (CI)
p = prevalence taken as 50% for maximum sample size calculation
q = 1-p
e = margin of error, 5%

The calculated minimum sample size was 385. However, 389 patients were included in the study. Data was collected on a predetermined proforma covering the relevant subjects of the study and the data were taken from the hospital records. Diagnosis of hepatic encephalopathy was made on the basis of history and physical examination. Factors like cognitive dysfunction, disturbed sleep/wake cycle, and personality changes were considered while diagnosing hepatic encephalopathy. Hepatic encephalopathy was then graded as per West Haven criteria into minimal, Grade I, Grade II, Grade III, and Grade IV.^5^

Data were entered and analyzed using Microsoft Excel 2016. Point estimate and 95% CI were calculated.

## RESULTS

Among 389 patients with chronic liver disease, hepatic encephalopathy was seen in 40 (10.28%) (7.26-13.30, 95% CI). The mean age for patients with HE was 55.27±13.52 years and 13 (32.50%) patients were from the age group 50-59 years (Table 1).

**Table 1:** Hepatic encephalopathy in different age groups (n= 40).

Age group (years)	n (%)
30-39	5 (12.50)
40-49	8 (20)
50-59	13 (32.50)
60-69	7 (17.50)
70-79	5 (12.50)
80-90	2 (5)

**Figure 1:**
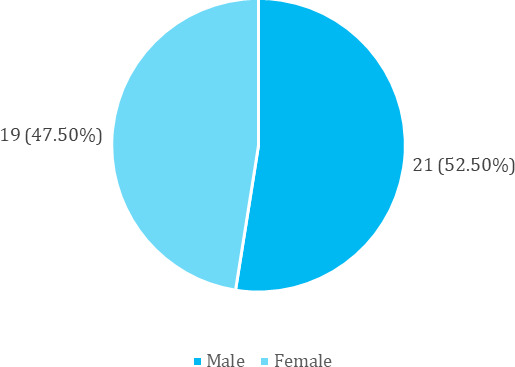
Gender-wise distribution (n= 40)

Hepatic encephalopathy was moreprevalent in males21 (52.50%) (Figure 1).

**Figure 2:**
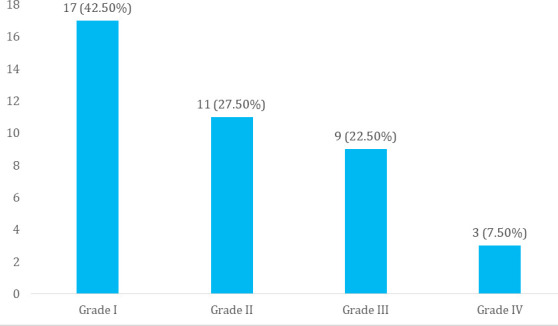
Grades of hepatic encephalopathy at presentation (n= 40).

Among patients with hepatic encephalopathy, 23 (57.5%) patients were found to have overt HE. Our study shows a greater number of patients classifying under Grade-I according to West Haven criteria i.e. 17 (42.5%), which falls under covert HE (Figure 2).

Among 40 patients, gastrointestinal bleed was seen in 18 (45%) (Table 2).

**Table 2:** Precipitating factors (n = 40).

Precipitating factors	n (%)
Gastrointestinal bleed	18 (45)
Infections	5 (12.50)
Constipation	5 (12.50)
Electrolyte imbalance	3 (7.50)
Others	7 (17.50)

## DISCUSSION

The prevalence of hepatic encephalopathy among patients with chronic liver disease was found to be 40 (10.28%) with Grade II HE being the most common overt HE. This is in concordance with a study conducted in Nepal, which had a prevalence of 14.90%.^2^ Additionally, we found covert HE was prevalent in 42.50% and overt HE in 57.50% of patients with HE. In contrast to our finding, another similar study showed covert HE was more common than overt HE with a prevalence of 58.30% and 41.70% respectively.^7^

According to our study, the prevalence of overt HE and covert HE among CLD patients is 5.91% and 4.37% respectively. On the contrary, western studies have shown that Overt HE is seen in 30%-45% of cirrhotic patients.^8^ Whereas, minimal HE or covert HE is seen in 20%-80% of cirrhotic patients, which is significantly higher than our findings.^9-13^

HE can also be subdivided into precipitated and nonprecipitated based on the presence of predisposing factors. Our study found gastrointestinal bleeding to be the most common precipitating factor of HE among CLD patients at 45% followed by infections at 12.50%, constipation at 12.50%, and electrolyte imbalance at 7.50%. This is similar to a study done at Manipal College of Medical Sciences and Teaching Hospital, Pokhara, Nepal which also showed gastrointestinal bleeding as the common precipitating factor.^14^ However, a study conducted in Hyderabad, India found infection (67%) as the most common factor followed by constipation (49%) and gastrointestinal bleeding (45%).^15^ These wide ranges of prevalence may exist because of the variation in diagnostic tests used for HE, and differences in the etiology and severity of HE that may differ based on the geographical location.

Our study is single-centered and the sample size is also small. Therefore, the results cannot be generalized in a larger population. The diagnostic tool, West Haven Criteria is a subjective tool, it has limited reliability for diagnosing HE that manifests with minimal alteration in mental and motor functions which may be missed. Therefore, additional neuropsychological and psychometric tests must be used as well. The sparse use of psychometric tests might be one of the reasons for lower prevalence of covert hepatic encephalopathy in our study.

## CONCLUSIONS

The prevalence of hepatic encephalopathy among patients with chronic liver disease in our study was lower than the studies done in similar settings. The reason behind this may be unrecognized minimal HE cases due to the lack of extensive use of psychometric tests that have greater sensitivity in detecting subtle changes in mental functions which are missed by common clinical bedside tests. Hence, easy-to-use psychometric tests like number connection test must be used routinely in our setting for better diagnostic accuracy which eventually will improve patient outcomes.
